# Supplementary material for: Prevalence, risk factors and consequences of newborns born small for gestational age: a multisite study in Nepal
Source: BMJ Paediatr Open. 2020 Mar 31;4(1):e000607. doi: 10.1136/bmjpo-2019-000607 (PMC7173954; doi:10.1136/bmjpo-2019-000607)
Supplement: Supplementary data [file bmjpo-2019-000607supp001.pdf]

ID NUMBER: ______________________

Form 2

NePeriQIP Client Exit Interview

Data Collector	Name…………………………………………………………………….. Code………………………………………………………………………

Data ID	Information		Write or circle where applicable	Notes
**PART A: BACKGROUND INFORMATION**				
201	Mother's first name			
202	Mother's last name			
203	Inpatient number			
204	Age of mother (completed years)			
205a	Caste			
205b	Ethnicity Code			
206	Address	District		
		Municipality		
		Ward		
207	Informed consent received?		Yes ……………………………………………… 1 No ……………………………………………… 0	
If no informed consent obtained, stop interview				
208	Contact number	Mobile	☐☐☐☐☐☐☐☐☐☐	
		Husband	☐☐☐☐☐☐☐☐☐☐	
		Landline	☐☐☐☐☐☐☐☐☐☐	
		Others	☐☐☐☐☐☐☐☐☐☐	

	Date (BS) (dd/mm/yyyy)	Signature
Form completed:		
Data entered into data base:		

Gautam Paudel P, *et al. BMJ Paediatrics Open* 2020; 4:e000607. doi: 10.1136/bmjpo-2019-000607

ID NUMBER: ______________________

209	What is your education level?	Illiterate………..………………..…..………………..….1<br>Able to read and write……………………………..2<br>Complete Primary………..……..…..……………...3<br>Completed Secondary….………............……...4<br>Started Higher……………………..……………....5		
210	Do you smoke?	Yes ………………………………………………………1<br>No ……………………………………………………… 0		If No, go to 213
211	If yes, how many cigarettes per day?	☐☐		
212	Do anyone residing in the same house smoke?	Yes ………………………………………………………1<br>No ……………………………………………………… 0		
213	**What type of fuel does your household mainly use for cooking?**			
	213a. *Electricity*	1	213d. *Biogas*	1
	213b. *LPG*	1	213e. *Kerosene*	1
	213c. *Natural gas*	1	213f. *Wood*	1
	213g. *No food cooked in household*	1		
	213h. *Others (specify)*			
214	In this household, what is food cooked on?	Open fire……………..…………………….….1<br>Stove ……………………………………..….…2<br>Chulo……………………………………………......3<br>Other (specify)…………..………..………….		
215	Does this (fire/stove/chulo/other) have a chimney, a hood, or neither of these?	Chimney…………………..………………..........1<br>Hood……………………………………..……….2<br>Neither……………………………………….…......3		
216	Is the cooking usually done in the house, in a separate building, or outdoors?	In the house…………………………..……….....1<br>Separate building…………………………….….2<br>Outdoors………………………………….............3<br>Other (specify)…………………………….….….		
217	Do you have a separate room that is used as a kitchen?	Yes…………………………………………..……...1<br>No……………………………………..…………...0		
218	Can you provide monthly family income?	…………………………………………(rupees)		

Gautam Paudel P, *et al. BMJ Paediatrics Open* 2020; 4:e000607. doi: 10.1136/bmjpo-2019-000607

ID NUMBER: ______________________

PART B: OBSTETRIC INFORMATION

**Part B1: Previous Obstetric History**			
219	Parity	Primipara (no previous children) ……….....1<br>Multipara (1-5 previous children)….……….2<br>Grand multipara (> 5 previous children)….3	
220	Number of previous pregnancies	☐☐	
221	Number of previous abortions (GA <22weeks)	☐☐	
222	Number of previous C-section	☐☐	
223	Number of previous still births (GA ≥22 weeks)	☐☐	
224	Number of previous live births	☐☐	
225	Number of previous neonatal deaths	☐☐	
**Part B2: Current Pregnancy History**			
226	Antenatal card available	Yes…………………………………………..1<br>No…………………………………………..0	
227	Current pregnancy	Single………………………………………..1<br>Multiple……………………………………..2	
228	Was current pregnancy planned? Was it at the right time in life?	Yes…………………………………………..1<br>No…………………………………………..0	If Yes, go to 230
229	If Not, did you want to wait until later or did you wish not to become pregnant?	Wait until later…………………………….1<br>Not become pregnant …………………….2<br>Don't know 98	
230	ANC check up by doctor/nurse/ANM	Yes…………………………………………..1<br>No…………………………………………..0	If No, go to 233
231	If Yes, how many?	☐☐	
232	Time for first ANC visit	First trimester (1-3 months pregnant)… …..1<br>Second trimester (4-6 months pregnant)….2<br>Third trimester (7-9 months pregnant)……3	

233	Delivery preparations	Yes	No	
	a. Did you plan where to deliver?	1	0	
	b. Did you plan for transport to delivery?	1	0	
	c. Did you save money for expenses?	1	0	
	d. Did you have two blood donors?	1	0	
	e. Any other preparations?	1	0	
234	Other preparations, please specify:			

Gautam Paudel P, *et al. BMJ Paediatrics Open* 2020; 4:e000607. doi: 10.1136/bmjpo-2019-000607

ID NUMBER: ________________________

PART C: DELIVERY				
235	Date of delivery (BS) (dd/mm/yyyy)	[ ][ ][ ][ ][ ][ ][ ][ ]		
		Single or Twin 1	Twin 2	
236	Sex of babies	Girl…………………1<br>Boy…………………..0	Girl…………………1<br>Boy…………………..0	
237	How did you deliver your babies?	Spontaneous vaginal…………………......1<br>Instrumental delivery……………………......2<br>Emergency CS………...................................3<br>Elective CS………….............................4		
238	Did you hear both of your babies cry immediately after birth?	Yes………………………………………..…………...1<br>No………………………….………………….……....0		If Yes, go to 241
239	Were the babies given any intervention to help the babies cry?	Yes……………………………………………………........1<br>No………………………….…………………..…… 0<br>Don't know……………………………………… 9		If No, go to 241
240	If yes, did the health provider explain to you what happened regarding the resuscitation?	Yes……………………………………………………........1<br>No………………………….…………………..…… 0<br>Don't know……………………………………… 9		
241	Were there any complications while delivering the babies?	Yes……………………………………………………........1<br>No………………………….…………………..…… 0<br>Don't know……………………………………… 9		
242	If Yes, what were the complications?	……………………………………………..(specify)		If No, go to 243
243	Have you stayed for at least 24 hours after an uncomplicated vaginal birth at the health facility?	Yes………………………………………..…………..1<br>No………………………….………………….……...0		
244	Did you have a companion of your choice during labour and child birth?	Yes………………………………………..…………...1<br>No………………………….…………………….…...0		
245	If Yes, who accompanied you?	………………………………………………………….		

Gautam Paudel P, *et al. BMJ Paediatrics Open* 2020; 4:e000607. doi: 10.1136/bmjpo-2019-000607

ID NUMBER: ______________________

PART D: ESSENTIAL NEWBORN CARE			
**Part D1: Nutrition and Breast Feeding**			
		Single or Twin 1	Twin 2
246	Were the babies breastfed before transfer to postnatal ward?	Yes………………………1<br>No……………………..…..0<br>Don't know…………….9	Yes………………………1<br>No……………………..…..0<br>Don't know…………….9
247	Did you have any difficulties to start breastfeeding? ***(If No, go to 249)***	Yes………………………1<br>No……………………..…..0	Yes………………………1<br>No……………………..…..0
248	If Yes, what difficulties?		
249	Did you receive breastfeeding counselling from a skilled health service provider before discharge?	Yes………………………1<br>No……………………..…..0	Yes………………………1<br>No……………………..…..0
250	Did you receive written or verbal information and counselling on exclusive breastfeeding until 6 complete months before discharge?	Yes………………………1<br>No……………………..…..0	Yes………………………1<br>No……………………..…..0
251	Did you receive counselling on supplementary feeding after 6 complete months before discharge?	Yes………………………1<br>No……………………..…..0	Yes………………………1<br>No……………………..…..0
252	Were the babies been given anything else than breast milk or medicines? ***(If No or Don't know, go to 254)***	Yes………………………1<br>No……………………..…..0<br>Don't know…………….9	Yes………………………1<br>No……………………..…..0<br>Don't know…………….9
253	If Yes, what was given?	Formula……………………1<br>Water or other fluids……2<br>Others (specify)………………………..	Formula……………………1<br>Water or other fluids……2<br>Others (specify)………………………..
254	Did you receive written or verbal information and counselling on nutrition and how to eat healthy?	Yes………………………1<br>No……………………..…..0	Yes………………………1<br>No……………………..…..0
**Part D2: Kangaroo Mother Care (KMC)**			
		Single or Twin 1	Twin 2
255	Were your babies born before the expected date of delivery or born too soon or too small?	Yes………………………1<br>No……………………..…..0<br>Don't know…………….9	Yes………………………1<br>No……………………..…..0<br>Don't know…………….9
256	How much did you babies weigh? (grams)	☐☐☐☐	☐☐☐☐
If babies not born too soon or too small or birth weight >2000 grams, go to Part D3!			

Gautam Paudel P, *et al. BMJ Paediatrics Open* 2020; 4:e000607. doi: 10.1136/bmjpo-2019-000607

ID NUMBER: ______________________

257	Did someone counsel you on ways to help your babies because they were born too soon or too small?	Yes………………………………………….……......1<br>No…………………………………….………..….0<br>Don't know…………………………………..… 9		
258	Did a health worker talk to about Kangaroo Mother Care (KMC)?	Yes………………………………………….……......1<br>No…………………………………….………..….0<br>Don't know…………………………………..… 9		If No or Don't know, go to 260
		Single or Twin 1	Twin 2	
259	If Yes, did your babies receive KMC? (Probe)	Yes…………………………...1<br>No……………………..…..0<br>Don't know……………….9	Yes…………………………...1<br>No……………………..…..0<br>Don't know……………….9	
**Part D3: Hygiene and temperature control**				
264	Did you receive written or verbal information and counselling on how to keep the babies warm?	Yes………………………………………….……......1<br>No…………………………………….………..….0<br>Don't know…………………………………..… 9		
		Single or Twin 1	Twin 2	
260	Have you kept your babies skin-to-skin contact immediately after birth? ***(If No or Don't know, go to 262)***	Yes…………………………...1<br>No……………………..…..0<br>Don't know……………….9	Yes…………………………...1<br>No……………………..…..0<br>Don't know……………….9	
261	If yes, how long? (hours)	☐☐	☐☐	
262	Were the newborns' body and head covered after birth?	Yes…………………………...1<br>No……………………..…..0<br>Don't know……………….9	Yes…………………………...1<br>No……………………..…..0<br>Don't know……………….9	
263	How were the babies kept warm on the first day of birth?	Wrapping………………….1<br>Skin-to-skin………..…….2<br>Incubator………………… 3<br><br>Others (specify)……… ….<br>Don't know……………...9	Wrapping………………….1<br>Skin-to-skin………..…….2<br>Incubator………………… 3<br><br>Others (specify)…………<br>Don't know……………...9	
265	How were the babies cleaned on the first day of birth?	Bathing with cold water….1<br>Bathing with warm water..2<br>Wiping with fabric………... 3<br>Not cleaned…………..……...4<br><br>Others (specify)……………<br>Don't know………………… 9	Bathing with cold water….1<br>Bathing with warm water..2<br>Wiping with fabric………... 3<br>Not cleaned…………..……...4<br><br>Others (specify)……………<br>Don't know………………… 9	
266	When were the babies first bathed? (24 hours clock)	☐☐☐☐	☐☐☐☐	
267	When was the vernix removed after birth? (24 hours clock)	☐☐☐☐	☐☐☐☐	

Gautam Paudel P, *et al. BMJ Paediatrics Open* 2020; 4:e000607. doi: 10.1136/bmjpo-2019-000607

ID NUMBER: ________________________

268	What was used to cut the umbilical cords?	New razor blade…………….1<br>Used razor blade…………… 2<br>Scissors………………………....3<br><br>Others (specify)……………<br>Don't know……………….... 9	New razor blade…………….1<br>Used razor blade…………… 2<br>Scissors………………………....3<br><br>Others (specify)……………<br>Don't know……………….... 9
269	Have you seen the umbilical stump?	Yes…………………………...1<br>No……………………….…..0<br>Don't know……………….9	Yes…………………………...1<br>No……………………….…..0<br>Don't know……………….9
270	Were the umbilical cord stumps covered?	Yes…………………………...1<br>No……………………….…..0<br>Don't know……………….9	Yes…………………………...1<br>No……………………….…..0<br>Don't know……………….9
271	Was anything applied to the umbilical stumps? ***(If No or Don't know, go to 275)***	Yes…………………………...1<br>No……………………….…..0<br>Don't know……………….9	Yes…………………………...1<br>No……………………….…..0<br>Don't know……………….9
272	If so, what was applied?		
273	Was chlorhexidine applied on the stump?	Yes…………………………...1<br>No……………………….…..0<br>Don't know……………….9	Yes…………………………...1<br>No……………………….…..0<br>Don't know……………….9
274	Who applied it?	Myself …. ………………..1<br>Health worker…………..2<br>Family member………..3<br>Others…………………………	Myself …. ………………..1<br>Health worker…………..2<br>Family member………..3<br>Others…………………………
275	Did you receive counselling on cord care?	Yes…………………………...1<br>No……………………….…..0	Yes…………………………...1<br>No……………………….…..0
276	Did you receive counselling on how to maintain hygiene (clean) of mother and babies?	Yes…………………………...1<br>No……………………….…..0	Yes…………………………...1<br>No……………………….…..0

Gautam Paudel P, *et al. BMJ Paediatrics Open* 2020; 4:e000607. doi: 10.1136/bmjpo-2019-000607

ID NUMBER: ________________________

Part D4: Danger signs				
277	Did the health worker counsel you on danger signs of mother during delivery and postnatal period?	Yes……………………………………………......1<br>No……………………………….…………...…..0<br>Don't know……………………………………... 9		If Don't know, probe
278	If Yes, what maternal danger signs did they counsel about? (multiple response)	Spontaneously mentioned	Mentioned after probing	
	a. Fever	1	1	
	b. Bleeding	1	1	
	c. Headache	1	1	
	d. Unconsciousness	1	1	
	e. Swelling	1	1	
	f. Abdominal pain	1	1	
	g. Foul smelling discharge	1	1	
	h. Others (specify			
279	Did the health workers counsel you on danger signs of the babies during delivery and postnatal period?	Yes……………………………………………......1<br>No……………………………….…………...…..0<br>Don't know……………………………………... 9		If Don't know, probe
280	If Yes, what newborn danger signs did they counsel about? (multiple response)	Spontaneously mentioned	Mentioned after probing	
	a. Fever	1	1	
	b. Unable to breastfeed	1	1	
	c. Fast breathing	1	1	
	d. Chest in drawing	1	1	
	e. Umbilical infection	1	1	
	f. Lethargy or unconscious	1	1	
	g. Vomiting	1	1	
	h. Hypothermia	1	1	
	i. Others (specify)			

Gautam Paudel P, *et al. BMJ Paediatrics Open* 2020; 4:e000607. doi: 10.1136/bmjpo-2019-000607

ID NUMBER: ______________________

PART E: DISCHARGE				
281	Did a medical doctor examine your baby when you were present?	Yes……………………………………………….1<br>No………………………………………………..0<br>Don't know……………………………………. 9		
282	Did a health worker examine your baby before discharge?	Yes……………………………………………….1<br>No………………………………………………..0<br>Don't know……………………………………. 9		
283	Before discharge, did you receive counselling on:	Yes	No	
	a. the care of the baby?	1	0	
	b. playing with baby?	1	0	
	c. birth spacing and family planning?	1	0	
	d. immunization schedule for the baby?	1	0	
	e. registering the birth of the baby?	1	0	
284	Were you given the opportunity to discuss any concerns and preferences?	Yes………………………………………………..1<br>No………………………………………………..0		
285	Were you adequately informed by the care provider about examinations, actions and decisions taken for your care throughout the hospital stay?	Yes………………………………………………..1<br>No………………………………………………..0		
286	Are you satisfied with the health education and information you received from health care providers?	Very satisfied……………………………………..5<br>Satisfied……………………………………… 4<br>Neither……………………………………………3<br>Dissatisfied…………………………………….2<br>Very dissatisfied………………………………1		
287	Are you satisfied with the degree of privacy during your stay in labour and child birth areas?	Very satisfied……………………………………..5<br>Satisfied……………………………………… 4<br>Neither……………………………………………3<br>Dissatisfied…………………………………….2<br>Very dissatisfied………………………………1		
288	Were you treated with respect and was your dignity preserved during your stay at the hospital?	Yes………………………………………………..1<br>No………………………………………………..0		
289	Did the health service meet your religious and cultural birthing practice needs?	Yes………………………………………………..1<br>No………………………………………………..0		
290	Were you or your newborn physically, verbally or sexually abused during labour or childbirth or after birth? Were you treated in a bad way?	Yes………………………………………………..1<br>No………………………………………………..0		If No or Don't know, go to 292

Gautam Paudel P, *et al. BMJ Paediatrics Open* 2020; 4:e000607. doi: 10.1136/bmjpo-2019-000607

ID NUMBER: ______________________

291	If Yes, what exactly happened? (allow multiple responses)	Pinched……………………..…………….…1<br>Slapped……………………..………………..2<br>Beaten…………………………………..……3<br>Episiotomy without anaesthesia……..…. 4<br>Tied to bed……………….……………………5<br>Verbally scolded……….…. ....6<br><br>Other (……………….……………......................	
292	Were you refused care because of inability to pay?	Yes……………………………………….……......1<br>No……………………………..……………..…..0<br>Don't know……………………………………... 9	
293	Were you asked by the health workers for extra money while at the hospital?	Yes……………………………………….……......1<br>No……………………………..……………..…..0<br>Don't know……………………………………... 9	
294	**How much did you pay for the services you received?**		
294a.	*Admission charge*	……………………………….……..(in rupees)	If no fees were paid for the services, go to 295
294b.	*Bed charge*	……………………………….……..(in rupees)	
294c.	*Laboratory diagnosis*	……………………………….……..(in rupees)	
294d.	*Doctor fees*	……………………………….……..(in rupees)	
294e.	*Medicines*	……………………………….……..(in rupees)	
294f.	*Others……………………………………………*	……………………………….……..(in rupees)	
294h.	*Others……………………………………………*	……………………………….……..(in rupees)	
295	**What were the additional expense you or your family had to bear for delivery?**		
295a.	*Transportation*	……………………………….……..(in rupees)	If no any additional expenses were paid, go to 296
295b.	*Lodging*	……………………………….……..(in rupees)	
295c.	*Food*	……………………………….……..(in rupees)	
295d.	*Others……………………………………………*	……………………………….……..(in rupees)	
296	Do you know about the '*Aama Karyakram*' or free maternity incentive scheme?	Yes…………………………………..………..1<br>No…………………………………………..……..0	
297	Did you receive transportation incentive?	Yes……………………………………….……......1<br>No……………………………..……………..…..0<br>Don't know……………………………………... 9	
298	Overall, how satisfied are you with the services?	Very satisfied……………..……………..…..5<br>Satisfied…………………………..……….. 4<br>Neither………………………………..……..3<br>Dissatisfied…………….....................……..2<br>Very dissatisfied………..………….............1	
299	Would you recommend a friend to deliver at this hospital?	Yes……………………………………….……......1<br>No……………………………..……………..…..0<br>Don't know……………………………………... 9	

Gautam Paudel P, *et al. BMJ Paediatrics Open* 2020; 4:e000607. doi: 10.1136/bmjpo-2019-000607
