# Supplementary material for: Prevalence, risk factors and consequences of newborns born small for gestational age: a multisite study in Nepal
Source: BMJ Paediatr Open. 2020 Mar 31;4(1):e000607. doi: 10.1136/bmjpo-2019-000607 (PMC7173954; doi:10.1136/bmjpo-2019-000607)
Supplement: Supplementary data [file bmjpo-2019-000607supp002.pdf]

Supplementary table 1 Variables included in the study

Variables		Definition	Categories
Outcome variable	Small for gestational age	Neonates whose birth weight was lower than 10th percentile for gestational age	Small for gestational age (SGA)
			Appropriate for gestational age (AGA)
Socio-demographic variables	Mother's age	Age of the mother in years	15-19 years
			20-34 years
			35 and above
	Education	Education level of mother	Illiterate (women who could not read or write)
			Literate (women who could read and write)
			Basic education (women whose education level was less than tenth grade)
			Secondary education and above (women whose education level was more than tenth grade)
	Ethnicity	Castes based on the hierarchical caste system of Nepal	Dalit<br>Janjati<br>Madhesi<br>Muslim<br>Chhetri/Brahmin<br>Others
	Smoking	Women who had history of smoking	Yes/No
	Passive smoking	Women living with someone who smoke in the same house at the time of data collection	Yes/No
	Types of fuel	Fuel used for the cooking purpose	Clean fuel (biogas/electricity/LPG/natural gas)
			Polluted fuel (kerosene/wood)
			Both clean and polluted
Obstetric variables	Parity	Total number of previous pregnancies	Nulliparous (never carried a pregnancy)
			Primiparous (1 birth)
			Multiparous (>2 births)
	Deliveries	Number of deliveries in women [for example single or more than one (twins, triplets)]	Single/Multiple
	Severe Anaemia	Women whose haemoglobin level is below 7g/dl	Yes/No
	Antepartum hemorrhage	Bleeding during pregnancy prior to the birth of baby	Yes/No
	ANC visit		Less than 4 ANC visits

		Number of ANC visit by women during pregnancy	4 or more ANC visits
	First ANC visit	Time of first ANC visit by women during pregnancy	First trimester/Second trimester/third trimester
	Delivery preparation	Women prepared regarding place to deliver, plan for transportation and financial	Yes/No
Neonatal variable	Sex of baby	Sex of baby born to women	Male/Female
